# Predictors and long-term outcome of seizures in human immuno-deficiency virus (HIV)-negative cryptococcal meningitis

**DOI:** 10.1186/s12883-014-0208-x

**Published:** 2014-10-13

**Authors:** Chih-Wei Hung, Wen-Neng Chang, Chia-Te Kung, Nai-Wen Tsai, Hung-Chen Wang, Wei-Che Lin, Chi-Ren Huang, Chih-Cheng Huang, Wan-Chen Tsai, Hsueh-Wen Chang, Yu-Jih Su, Yu-Jun Lin, Ben-Chung Cheng, Ya-Ting Chang, Chih-Min Su, Cheng-Hsien Lu

**Affiliations:** Department of Emergency Medicine, Chang Gung Memorial Hospital-Kaohsiung Medical Center, Chang Gung University College of Medicine, Kaohsiung, Taiwan; Department of Neurology, Chang Gung Memorial Hospital-Kaohsiung Medical Center, Chang Gung University College of Medicine, Kaohsiung, Taiwan; Department of Neurosurgery, Chang Gung Memorial Hospital-Kaohsiung Medical Center, Chang Gung University College of Medicine, 123, Ta Pei Road, Niao Sung Hsiang, Kaohsiung, Taiwan; Department of Radiology, Chang Gung Memorial Hospital-Kaohsiung Medical Center, Chang Gung University College of Medicine, Kaohsiung, Taiwan; Department of Biological Science, National Sun Yat-Sen University, Kaohsiung, Taiwan; Department of Internal Medicine, Chang Gung Memorial Hospital-Kaohsiung Medical Center, Chang Gung University College of Medicine, Kaohsiung, Taiwan

**Keywords:** Outcome, Risk factors, Seizures, HIV-negative cryptococcal meningitis

## Abstract

**Background:**

Seizures are one of the most important neurologic complications of human immuno-deficiency virus (HIV)-negative cryptococcal meningitis. A better understanding of the risk associated factors can help predict those who will require treatment.

**Methods:**

This 22-year retrospective study enrolled 180 patients. Prognostic variables independently associated with seizures or fatality were analyzed using stepwise logistic regression.

**Results:**

Twenty-eight patients with HIV-negative cryptococcal meningitis had seizures, including 13 with early seizures and 15 with late seizures. The mean time interval from HIV-negative cryptococcal meningitis to first seizure in the early and late seizure groups were 1.5 and 51.4 days, respectively. Nine out of the 28 cases (32%) occurred within 24 hours of presentation. The overall mortality rate was 54% (15/28) and two patients progressed to epilepsy.

**Conclusions:**

Patients with seizure have worse outcomes and longer hospitalization. Most first seizures occur within one year after the diagnosis of HIV-negative cryptococcal meningitis.

## Background

Despite advances in modern neurosurgical techniques, new anti-fungal agents, and powerful imaging technologies, cryptococcal meningitis remains a potentially fatal central nervous system (CNS) infection [1-3]. Seizures are important neurologic complications that may occur early or late in both HIV-negative and HIV-infected cryptococcal meningitis [1-6]. The reported incidence varies from 7.6% to 28.6% in different series [2,3]. To date, only one clinical research has focused specifically on seizures complicating HIV-negative cryptococcal meningitis [3]. The indications and duration of seizure treatment remain controversial [7], and the potential adverse effects of anti-epileptic drugs (AEDs) are a concern. Thus, treatment is mainly symptomatic.

The present hospital-based study may provide accurate information on the relative frequency of seizure sub-types and their effects on fatality and on neurologic sequelae, and their relationship with underlying cerebral pathologic lesions. Because of the benefits of AEDs in reducing morbidity after seizures, there is a need for better delineation of potential prognostic factors and outcomes in hospitalized patients with HIV-negative cryptococcal meningitis who should receive treatment. This study analyzed the clinical features, neuro-imaging findings, clinical scores, and measurements to determine potential risk factors predictive of seizures in HIV-negative cryptococcal meningitis.

## Methods

### Study population

The medical records of patients with HIV-negative cryptococcal meningitis admitted to Kaohsiung Chang Gung Memorial Hospital between 1986 and 2007 were reviewed for blood cultures, microbiologic records, and neuro-imaging findings using pre-existing standardized evaluation forms. Kaohsiung Chang Gung Memorial Hospital is a 2482-bed acute-care teaching hospital that provides both primary and tertiary referral care.

### Diagnostic criteria of HIV-negative cryptococcal meningitis

Patients with HIV-negative cryptococcal meningitis were included if they had: (1) positive isolation of Cryptococcus neoformans (C. neoformans) in one or more cerebro-spinal fluid (CSF) cultures, positive CSF cryptococcal antigen titer, or positive CSF India ink and clinical features of meningitis; or (2) isolation of C. neoformans in blood culture, with clinical presentations of meningitis and typical CSF features [1].

Patients were excluded if they had a history of seizures; pre-existing neurologic conditions with various neurologic deficits (e.g. stroke, head trauma, and hypoxic encephalopathy); or regularly took AEDs for epilepsy or other clinical indications (e.g. trigeminal neuralgia or neuropathic pain). Thus, only 180 of 185 patients were enrolled for analysis.

### Definition of seizures

Seizures were classified according to the recommendations of the International League against Epilepsy [8]. Status epilepticus (SE) was defined as a continuous behavioral seizure activity or repetitive seizures without full recovery of neurologic function between seizures, lasting longer than 30 minutes [9]. Seizure was defined according to those used in previous studies [10-12]. Seizures occurring after HIV-negative cryptococcal meningitis were causally related to the HIV-negative cryptococcal meningitis itself. A provoked (acute symptomatic) seizure was one that occurred in close temporal relation with HIV-negative cryptococcal meningitis, which was the presumed etiology [10-12]. In contrast, an unprovoked seizure was a seizure occurring in the absence of one or more precipitating factors, including events in patients with previously stable (non-progressing) HIV-negative cryptococcal meningitis [10-12].

Epilepsy was the occurrence of repeated unprovoked seizures [5,10-12]. Based on seizures onset in relation to the clinical ictus of HIV-negative cryptococcal meningitis, patients with seizures were divided into two sub-types. Early seizures were those occurring within two weeks of the infection, whereas late seizures were those occurring after two weeks.

### Study protocol

All of the patients underwent brain computed tomography (CT) scan at the emergency room. Follow-up brain CT scans and/or magnetic resonance imaging (MRI) were performed if there was clinical deterioration, including acute onset focal neurologic deficits, seizures or status epilepticus, progressively disturbed consciousness, and for post-neurosurgical procedure. Hydrocephalus was judged retrospectively by dilated temporal horn of the ventricle without obvious brain atrophy and/or an Evan’s ratio >0.3 on initial CT scan. The Evan’s ratio was the ratio of the ventricular width of the bilateral frontal horn to the maximum biparietal diameter [13,14].

The hospital’s standard protocol for HIV-negative cryptococcal meningitis was to administer AEDs only to those with acute symptomatic seizures. Prophylactic AED therapy was not given to asymptomatic patients in the acute stage. The AEDs were administered to patients with HIV-negative cryptococcal meningitis during hospitalization and were discontinued if there were no unprovoked seizures on follow-up.

The follow-up period was terminated by death or by the end of the study (December 2007). Most patients were followed-up at the out-patient department after discharge, while others were interviewed by telephone to identify neurologic outcomes. The frequency of seizures was determined by a Seizure Frequency Scoring System, which was slightly modified from Engel et al. [15] and a previous study [16]. Good outcome was defined as survival while poor outcome was defined as fatality during the follow-up period. The hospital’s Institutional Review Committee on Human Research approved the study protocol.

### Anti-fungal therapy

In the study hospital, anti-fungal therapy in patients with HIV-negative cryptococcal meningitis was started according to the preference of the attending physician [1,17-18]. The patients were divided into three major groups: Group I received Amphotericin B intravenously, with or without 5-flucytosine, for 6–10 weeks, for a total intravenous dose of 2–3 g; Group II received Amphotericin B with fluconazole 300–400 mg intravenously in the first 2–3 weeks, before treatment was switched to oral fluconazole alone (300–400 mg daily for 10 weeks); and Group III, received Fluconazole 400–800 mg intravenously daily during the first 2 weeks and then switched to an oral dose of 300–400 mg daily for the duration of primary therapy.

### Statistical analysis

Two separate statistical analyses were performed. First, the demographic data between the good and poor outcome groups were compared. Categorical variables were compared using the Chi-square test or Fischer’s exact test, as appropriate. Continuous variables within two groups were compared using the independent *t*-test for parametric data and the Mann–Whitney *U* test for non-parametric data. Second, to determine risk factors independently associated with seizures or fatality, the clinical and laboratory parameters with a p value <0.05 in univariate analysis were entered in a logistic regression model using a stepwise entry system. All statistical analyses were conducted using the SAS software package, version 13.0 (2002, SAS Statistical Institute, Cary, North Carolina).

## Results

### Baseline data of the study patients

The 180 patients (age, 18–88 years) with HIV-negative cryptococcal meningitis included 110 males and 70 females (mean age, 50.2 and 56.5 years, respectively). Their characteristics in terms of seizures (Table 1) revealed that 28 had seizures.

**Table 1:** **Characteristics of patients with HIV-negative cryptococcal meningitis in terms of seizures (n = 180)**

	**With seizures** ^**§**^			**Without seizures**
	**Early seizures**	**Late seizures**	***p*** **value**	
	**n = 13 (%)**	**n = 15 (%)**		**n = 152 (%)**
Mean age, years	47.3 ± 17.3	52.1 ± 25.0	0.32	53.2 ± 17.9
Sex (male/female)	7/6	9/6	0.74	92/60
Mean GCS on presentation	11.5 ± 3.9	12.1 ± 4.4	0.71	13.1 ± 3.1
Clinical features				
Fever/chills	8 (61.5)	13 (86.6)	0.20	89 (58.5)
Headache	9 (69.2)	9 (60)	0.71	69 (45.3)
Disturbed consciousness	8 (61.5)	8 (53.3)	0.66	61 (40.1)
Visual disturbance	2 (15.3)	5(33.3)	0.61	26 (17.1)
Hearing impairment	1 (7.6)	1 (6.6)	1.0	4 (2.6)
Hyponatremia	3 (23.1)	2 (13.3)	0.64	21 (13.8)
Outcome				
Death	5 (38.4)	10 (66.6)	0.14	42 (27.6)
Mean length of hospitalization (days)	58.0 ± 38.9	89.3 ± 62.6	0.08	56.9 ± 49.3

Abbreviations: HIV, human immuno-deficiency virus; GCS, Glasgow Coma Scale; SD, standard deviation; IQR, inter-quartile range.

Categorical data is presented as %.

^§^Patients with early and late seizure were compared.

### Neuro-imaging findings

The neuro-imaging findings of the HIV-negative cryptococcal meningitis patients with or without seizures were listed in Table 2. Hydrocephalus, gyral enhancement, basal cistern effacement, and cerebral infarction were the four most common findings in the seizure group, while hydrocephalus, dilated Virchow-Robin space, and gyral enhancement were the three most common findings in the non-seizure group.

**Table 2:** **Neuro-imaging findings**

	**Seizures after cryptococcal meningitis** ^**§**^			**Without seizures**
	**Early seizures**	**Late seizures**	***p*** **value**	
	**n = 13 (%)**	**n = 15 (%)**		**n = 152 (%)**
Hydrocephalus	7 (53.8)	6 (40.0)	0.743	51 (33.5)
Dilated Virchow-Robin space	1 (7.6)	1 (6.6)	1.0	13 (8.5)
Gyral enhancement	3 (23.0)	2 (13.3)	1.0	12 (7.8)
Basal cistern effacement	1 (7.7)	3 (20.0)	0.464	11 (7.2)
Pseudo-cyst	0	0	1.0	11 (7.2)
Cerebral infarction	0	4 (26.7)	0.644	10 (6.5)
Mass lesions	0	2 (13.3)	1.0	10 (6.5)

^§^Categorical data of neuroimaging findings between early seizure and late seizure were compared using Chi-square test or Fischer’s exact test.

Categorical data is presented as %.

### Clinical characteristics and seizure outcomes

**Figure 1:**
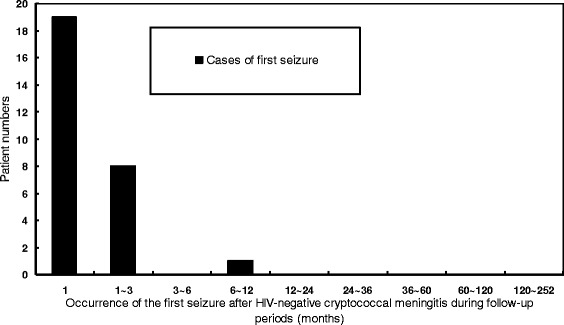
**The frequency distribution of patients with first seizures after HIV-negative cryptococcal meningitis during the study period (months).**

Regarding seizure onset in relation to the clinical ictus of HIV-negative cryptococcal meningitis, 13 had early seizures and 15 had late seizures (Table 1). The mean time interval from HIV-negative cryptococcal meningitis to first seizure in the early and late seizure groups were 1.5 and 51.4 days, respectively (Figure 1). Nine of the 28 cases (32%) occurred within 24 hours of presentation.

As regards seizure sub-types among the 28 cases, 18 were generalized tonic-clonic (64.3%, 18/28), 5 were focal seizures with secondary generalized seizures (17.8%, 5/28), three were myoclonic seizures (10.7%, 3/28), one was simple partial seizure (3.6%, 1/28), and the remaining one was complex partial seizure (3.6%, 1/28). Three (10.7%, 3/28) of the 28 cases progressed to status epilepticus.

In terms of treatment, 22 were treated with phenytoin alone, three with valproic acid alone, and the remaining three with both valproic acid and phenytoin. The mean duration of AED therapy for those who were discharged on AEDs was 12.9 ± 7.3 months. None of the 28 patients had drug-related complications.

The mean duration of hospitalization for the seizure and non-seizure groups was 77.54 ± 56.14 and 56.9 ± 5.29 days, respectively (p = 0.05). Fifteen (53.6%, 15/28) died in the acute phase of HIV-negative cryptococcal meningitis, while 13 (46.4%, 13/28) survived. In terms of seizure control after discharge among the 13 survivors, 5 (38.4%, 5/13) were seizure-free without AED therapy, 6 (46.2%, 6/13) were seizure-free under AED therapy, one (7.7%, 1/13) had 4–11 seizures per year, and the remaining one (7.7%, 1/13) had 1–3 seizures per month.

Twenty-three (12.8%, 23/180) cases were lost to follow-up after discharge during the study period. In total, 123 of 180 (68.3%, 123/180) patients survived. The fatality rate in the seizure and non-seizure groups was 54% (15/28) and 12% (42/152), respectively. The mean age at onset, median days of hospitalization, and median GCS on admission between the two groups were listed in Table 3.

**Table 3:** **Comparison of baseline clinical features and neuro-imaging findings between HIV-negative cryptococcal meningitis patients with and those without seizures**

	**Without seizures n = 152 (%)**	**With seizures n = 28 (%)**	***p*** **value**	**OR**	**95% CI**
Sex (Male/female)	92/58	16/12	0.76		
Mean age at onset	53.18 ± 17.86	49.86 ± 21.48	0.446		
Mean GCS on presentation	13.05 ± 3.11	11.79 ± 4.10	0.064		
Underlying diseases					
Diabetes mellitus	27 (17.8)	7 (25.0)	0.369	1.543	0.596-3.995
Pulmonary TB	8 (5.3)	4 (14.2)	0.095	3	0.838-10.743
Systemic lupus erythematosus	7 (4.6)	1 (3.6)	1	0.767	0.091-6.489
Liver cirrhosis	9 (5.9)	4 (14.3)	0.123	2.648	0.755-9.286
Chronic alcoholism	3 (2.0)	0	1	0.842	0.79-0.897
Autoimmune diseases	4 (2.6)	1 (3.6)	0.575	1.37	0.147-12.736
Hematological diseases	7 (4.6)	1 (3.6)	1	0.767	0.091-6.489
Malignance	10 (6.6)	1 (3.6)	1	0.526	0.065-4.279
Iatrogenic Cushing syndrome	12 (7.9)	1 (3.6)	0.695	0.432	0.054-3.463
Clinical features					
Fever/chills	89 (58.6)	21 (75.0)	0.101	2.124	0.851-5.298
Headache	69 (45.4)	18 (64.3)	0.066	2.165	0.938-4.997
Disturbed consciousness	61 (40.1)	16 (57.1)	0.095	1.989	0.88-4.497
Visual disturbance	19 (12.5)	7 (25.0)	0.084	2.333	0.875-6.225
Hearing impairment	4 (2.6)	2 (7.1)	0.235	2.846	0.496-16.342
Neuro-imaging findings at presentation					
Hydrocephalus	74 (48.7)	16 (57.1)	0.411	1.405	0.623-3.170
Cerebral infarction	14 (9.2)	4 (14.2)	0.489	1.643	0.498-5.415
Gyral enhancement	13 (8.6)	6 (21.4)	0.042	2.916	1.003-8.474
Basal cistern effacement	10 (6.6)	1 (3.6)	1	0.526	0.065-4.279
Pseudo-cyst	7 (4.6)	4 (14.2)	0.071	3.452	0.939-12.696
Dilated Virchow-Robin space	10 (6.6)	3 (10.7)	0.43	1.704	0.438-6.629
Mass lesions	8 (5.3)	2 (7.1)	0.656	1.385	0.278-6.891
Peripheral blood testing					
Leukocytosis	35 (23.0)	10 (35.7)	0.154	1.857	0.786-4.390
Leucopenia	2 (1.3)	0	1	0.843	0.791-0.898
Thrombocytopenia	12 (7.9)	6 (21.4)	0.028	3.182	1.083-9.352
Hyponatremia	21 (13.8)	5 (17.8)	0.576	1.356	0.465-3.958
CSF study					
Opening pressure (mmH2O)	305 ± 80.9	387.3 ± 154.1	0.09		
Sugar (mg/dl)	39.04	41.16	0.766		
Total Protein (mg/dl)	203.78	285.95	0.282		
Lactate (mg/dl)	51.56	61.67	0.217		
CSF cryptococcal antigen titer					
≥1:1024	73 (48.0)	16 (57.1)	0.375	1.44	0.64-3.25
<1:1024	79 (52.0)	12 (42.9)			
Outcome					
Median (IRQ) hospitalization days	56.9 ± 45.29	77.54 ± 56.14	0.050		

*Abbreviation: HIV* human immuno-deficiency virus.

Categorical data is presented as %.

### Risk factors and outcome of epilepsy and fatality

Comparisons of clinical features and neuro-imaging findings between the seizure and non-seizure groups were listed in Table 3. Statistical analysis between the two patient groups revealed that only thrombocytopenia (p = 0.028) and neuro-imaging findings at presentation showing gyral enhancement (P = 0.042) were significant variables. After stepwise logistic regression analysis, both thrombocytopenia (*p* = 0.035, 95% CI: 1.09-9.76) and neuro-imaging findings at presentation showing gyral enhancement (*p* = 0.048, 95% CI 1.01-8.873) were independently associated with seizures.

Comparisons of clinical features and neuro-imaging findings between survivors and non-survivors after a minimum of 5 years of follow-up were listed in Table 4. Statistical analysis between the two groups revealed that mean GCS on admission (*p* < 0.001), presence of underlying diseases as liver cirrhosis (*p* = 0.004) and iatrogenic Cushing syndrome (*p* = 0.016), and the presence of seizures (*p* = 0.007) and CSF cryptococcal antigen titer ≥1:1024 and lactate level (*p* = 0.015) were all significant variables. Significant univariate factors used in the stepwise logistic regression were mean GCS on admission, presence of underlying diseases (liver cirrhosis and iatrogenic Cushing syndrome), seizures, and CSF cryptococcal antigen titer (≥1:1024 or not) and lactate level.

**Table 4:** **Risk factors for fatality in HIV-negative cryptococcal meningitis**

	**Non-fatality n = 123 (%)**	**Fatality n = 57 (%)**	***p*** **value**	**OR**	**95% CI**
Sex (Male/female)	76/45	32/25	0.435		
Mean age at onset	50.89 ± 18.8	56.49 ± 17.16	0.058		
Mean GCS on presentation	13.65 ± 12.35	11.12 ± 4.30	<0.0001		
Underlying diseases					
Diabetes mellitus	20 (16.2)	14 (24.6)	0.186	1.677	0.776-3.622
Pulmonary TB	8 (6.5)	4 (7.0)	1	1.085	0.313-3.762
Systemic lupus erythematosus	7 (5.7)	1 (1.8)	0.439	0.296	0.036-2.464
Liver cirrhosis	4 (3.3)	9 (15.8)	0.004	5.578	1.639-18.98
Chronic alcoholism	1 (0.8)	2 (3.5)	0.236	4.436	0.394-49.964
Autoimmune diseases	2 (1.6)	3 (5.2)	0.328	3.361	0.546-20.697
Hematologic diseases	6 (4.9)	2 (3.5)	1	0.709	0.139-3.627
Malignance	6 (4.9)	5 (8.8)	0.31	1.875	0.548-6.421
Iatrogenic Cushing syndrome	5 (4.0)	8 (14.0)	0.016	3.853	1.201-12.364
Clinical features					
Fever/chills	68 (55.3)	42 (73.7)	0.018	2.265	1.138-4.508
Headache	60 (48.8)	27 (47.4)	0.86	0.945	0.504-1.772
Disturbed consciousness	44 (35.8)	33 (57.9)	0.005	2.469	1.299-4.692
Visual disturbance	19 (15.4)	7 (12.3)	0.574	0.766	0.302-1.942
Hearing impairment	4 (3.3)	2 (3.5)	1	1.082	0.192-6.085
Seizure	13 (10.6)	15 (26.3)	0.007	3.022	1.326-6.885
Neuro-imaging findings at presentation					
Hydrocephalus	59 (48.0)	31 (54.4)	0.423	1.293	0.689-2.428
Cerebral infarction	12 (9.8)	6 (10.5)	0.873	1.088	0.387-3.062
Gyral enhancement	13 (10.6)	6 (10.5)	0.996	0.995	0.358-2.768
Basal cistern effacement	5 (4.0)	6 (10.5)	0.092	2.776	0.81-9.513
Pseudo-cyst	8 (6.5)	3 (5.3)	1	0.799	0.204-3.129
Dilated Virchow-Robin space	9 (7.3)	4 (7.0)	1	0.956	0.282-3.245
Mass lesions	6 (4.9)	4 (7.0)	0.728	1.472	0.399-5.433
Peripheral blood testing					
Leukocytosis	28 (22.8)	17 (29.8)	0.309	1.442	0.711-2.924
Leucopenia	2 (1.6)	0	1	0.68	0.615-0.752
Thrombocytopenia	9 (7.3)	9 (15.8)	0.078	2.375	0.888-6.351
Hyponatremia	17 (13.8)	9 (15.8)	0.727	1.169	0.486-2.81
CSF study					
Opening pressure (mmH2O)	356.7 ± 143.6	379.9 ± 149.3	0.679		
Sugar (mg/dl)	40.77 ± 34.11	35.69 ± 27.80	0.390		
Total Protein (mg/dl)	210.30 ± 360.94	235.75 ± 339.47	0.688		
Lactate (mg/dl)	48.76 ± 34.90	65.38 ± 40.92	0.015		
CSF cryptococcal antigen titer					
≥1:1024	48 (39.0)	41 (71.9)	<0.001	4.0	2.03-7.92
<1:1024	75 (61.0)	16 (28.1)			
Outcome					
Median (IRQ) hospitalization days	63.32 ± 49.39	48.44 ± 38.43	0.107		

*Abbreviation: HIV* human immuno-deficiency virus.

Categorical data is presented as %.

After analysis, only mean GCS on admission (OR: 0.835, 95% CI, 0.737-0.946; *p* = 0.005), presence of acute seizures (OR: 3.683, 95% CI: 1.332-10.18; *p* = 0.012), presence of underlying diseases such as liver cirrhosis (OR: 6.580, 95% CI: 1.348-32.12; *p* = 0.02) and iatrogenic Cushing syndrome (OR: 4.574, 95% CI: 1.185-17.66; *p* = 0.027) and CSF cryptococcal antigen titer ≥1:1024 (OR: 3.614, 95% CI: 1.093-6.247; *p* = 0.031) were independently associated with fatality. Any reduction of one point of GCS increased the fatality rate by 17%.

## Discussion

The frequency of seizures after HIV-negative cryptococcal meningitis is variously estimated at 7.6-28.6% [2,3]. To date, this is the largest study to show long-term outcomes of seizures among patients with HIV-negative cryptococcal meningitis. In this study, seizures occur in 28 out of 180 patients (15.6%), including early seizures in 13 (7.2%) and late seizures in 15 (8.3%). Among the 28 with seizure, two progressed to epilepsy.

### Major findings

The present study examined the predictive factors and outcomes of seizures in patients with HIV-negative cryptococcal meningitis and produced two major findings. First, patients with seizure had longer mean length of hospitalization. Lower mean GCS on admission and the presence of seizures were independently associated with fatality, and any reduction of one point of GCS increased the fatality rate by 17%. Second, seizures in patients with HIV-negative cryptococcal meningitis might have delayed manifestations although most seizures occur during the acute phase of the meningitis. None of the patients had a first seizure occurring more than one year after the diagnosis of HIV-negative cryptococcal meningitis. The occurrence of seizures was commonly observed within 24 hours of presentation in one study [3], as well as in the present study, and accounted for 100% (8/8) and 32% (9/28) of cases, respectively.

The use of prophylactic AED therapy for preventing seizures in patients with HIV-negative cryptococcal meningitis remains unclear. A possible benefit is the reduction in morbidity after seizures. At present, no formal recommendations can be made regarding the use of prophylactic AEDs for patients with HIV-negative cryptococcal meningitis based on currently available well-designed randomized controlled trials. Phenytoin is the most widely used AED in this study and there are no observed adverse effects related to its use.

In this study, lower mean GCS on admission, presence of acute seizures, presence of underlying diseases like liver cirrhosis and iatrogenic Cushing syndrome, and CSF cryptococcal antigen titer ≥1:1024 are independently associated with fatality. The CSF cryptococcal-antigen titer >1:1024 may imply severe infection. It is also a significant prognostic factor in both HIV-negative and HIV-positive cryptococcal meningitis [1,19]. There is a striking correlation between initial consciousness level and therapeutic outcome, and this is consistent with findings of other reports [20,21]. The presence of underlying diseases such as liver cirrhosis and iatrogenic Cushing syndrome is also a well-known predisposing factor of poor outcome [22,23].

Seizures complicating HIV-negative Cryptococcal meningitis have worse outcomes and longer hospitalization. There may be several reasons for the increased mortality and morbidity in patients with seizures. For instance, patients are commonly associated with underlying medical conditions. Moreover, later neurologic morbidity is attributable to hypoxia, lactic acidosis, increased intra-cranial pressure (IICP), and autonomic dysfunction.

### Study limitations

First, this was a retrospective analysis and therefore subject to bias of unmeasured factors (e.g. possible reporting bias due to patient selection presented to the hospital). It was also not possible to assess the effects of prophylactic AEDs after the acute stage of HIV-negative cryptococcal meningitis. Second, patients who took AEDs for epilepsy or other clinical indications (e.g. trigeminal neuralgia or neuropathic pain) or those with pre-existing neurologic deficits were excluded. Thus, there was continued uncertainty in assessing the incidence of unprovoked seizures after HIV-negative cryptococcal meningitis in non-selected patients. Third, the impact of surgical intervention for hydrocephalus on the frequency of seizures was not clear. Surgical interventions might cause potential brain insults and lead to unprovoked seizures during follow-up. Lastly, most patients in this study were treated with AEDs after their first acute symptomatic seizure, in accordance with the study protocol. Thus, the findings might underestimate the “true” frequency of seizures associated with the “natural history” of untreated unprovoked seizures.

## Conclusions

Among patients with HIV-negative cryptococcal meningitis, those with seizure have worse outcome and longer hospitalization. Most first seizures occur within one year of diagnosis of meningitis. Thus, prophylactic treatment, if considered, should not be performed for a period of more than one year.

### Ethical approval

The study was approved by Chang Gung Memorial Hospital’s Institutional Review Committee on Human Research.

## References

[1] Lu CH, Chang WN, Chang HW, Chuang YC (1999). The prognostic factors of cryptococcal meningitis in HIV-negative patients. J Hosp Infect.

[2] Zhu LP, Wu JQ, Xu B, Ou XT, Zhang QQ, Weng XH (2010). Cryptococcal meningitis in non-HIV-infected patients in a Chinese tertiary care hospital, 1997–2007. Med Mycol.

[3] Tiamkao S, Sawanyawisuth K, Chotmongkol V (2007). Seizure in non-HIV cryptococcal meningitis. J Med Assoc Thai.

[4] Boulware DR, Meya DB, Bergemann TL, Wiesner DL, Rhein J, Musubire A, Lee SJ, Kambugu A, Janoff EN, Bohjanen PR (2010). Clinical features and serum biomarkers in HIV immune reconstitution inflammatory syndrome after cryptococcal meningitis: a prospective cohort study. PLoS Med.

[5] Seboxa T, Alemu S, Assefa A, Asefa A, Diro E (2010). Cryptococcal meningitis in patients with acquired immuno-deficiency syndrome in pre-HAART era at Gondar College of Medical Sciences Hospital north-west Ethiopia. Ethiop Med J.

[6] Kisenge PR, Hawkins AT, Maro VP, McHele JP, Swai NS, Mueller A, Houpt ER (2007). Low CD4 count plus coma predicts cryptococcal meningitis in Tanzania. BMC Infect Dis.

[7] Perfect JR, Dismukes WE, Dromer F, Goldman DL, Graybill JR, Hamill RJ, Harrison TS, Larsen RA, Lortholary O, Nguyen MH, Pappas PG, Powderly WG, Singh N, Sobel JD, Sorrell TC (2010). Clinical practice guidelines for the management of cryptococcal disease: 2010 update by the Infectious Diseases Society of America. Clin Infect Dis.

[8] Commission on Classification and Terminology of the International League against Epilepsy (1981). Proposal for revised clinical and electroencephalographic classification of epileptic seizures. Epilepsia.

[9] Treiman DM (1995). Electro-clinical features of status epilepticus. J Clin Neurophysiol.

[10] Bladin CF, Alexandrov AV, Bellavance A, Bornstein N, Chambers B, Cote R (2000). Seizures after stroke: a prospective multi-center study. Arch Neurol.

[11] Passero S, Rocchi R, Rossi S, Ulivelli M, Vatti G (2002). Seizures after spontaneous supratentorial intra-cerebral hemorrhage. Epilepsia.

[12] Yang TM, Lin WC, Chang WN, Ho JT, Wang HC, Tsai NW, Shih YT, Lu CH (2009). Predictors and outcome of seizures after spontaneous intra-cerebral hemorrhage. J Neurosurg.

[13] Liliang PC, Liang CL, Chang WN, Chen HJ, Su TM, Lu K, Lu CH (2003). Shunt surgery for hydrocephalus complicating cryptococcal meningitis in human immuno-deficiency virus-negative patients. Clin Infect Dis.

[14] Liliang PC, Liang CL, Chang WN, Lu K, Lu CH (2002). Use of ventriculo-peritoneal shunts to treat uncontrollable intracranial hypertension in patients who have cryptococcal meningitis without hydrocephalus. Clin Infect Dis.

[15] Engel J, Van Ness PC, Rasmussen TB, Engel J (1993). Outcome with respect to epileptic seizures. Surgical Treatment of Epilepsies.

[16] Wang HC, Chang WN, Chang HW, Ho JT, Yang TM, Lin WC, Chuang YC, Lu CH (2008). Factors predictive of outcome in post-traumatic seizures. J Trauma.

[17] Saag MS, Graybill RJ, Larsen RA, Pappas PG, Perfect JR, Powderly WG, Sobel JD, Dismukes WE (2000). Practice guidelines for the management of cryptococcal disease: Infectious Diseases Society of America. Clin Infect Dis.

[18] Lee CH, Chang TY, Liu JW, Chen FJ, Chien CC, Tang YF, Lu CH (2012). Correlation of anti-fungal susceptibility with clinical outcomes in patients with cryptococcal meningitis. BMC Infect Dis.

[19] Anekthananon T, Manosuthi W, Chetchotisakd P, Kiertiburanakul S, Supparatpinyo K, Ratanasuwan W, Pappas PG, Filler SG, Kopetskie HA, Nolen TL, Kendrick AS, Larsen RA, BAMSG 3–01 Study Team (2011). Predictors of poor clinical outcome of cryptococcal meningitis in HIV-infected patients. Int J STD AIDS.

[20] Seaton RA, Naraqi S, Wembri JP, Warrell DA (1996). Predictors of outcome in Cryptococcus neoformans var. gattii meningitis. QJM.

[21] Saag MS, Powderly WG, Cloud GA, Robinson P, Grieco MH, Sharkey PK, Thompson SE, Sugar AM, Tuazon CU, Fisher JF, Hyslop N, Jacobson JM, Hafner R, Dismukes WE, NIAID mycoses study group and AIDS clinical trials group (1992). Comparison of amphotericin B with fluconazole in the treatment of acute AIDS-associated cryptococcal meningitis. N Engl J Med.

[22] Zhong YH, Tan F, Li M, Liu J, Wang X, Yuan Y, Zhong XF, Peng FH (2014). Comparisons of presentations and outcomes of cryptococcal meningitis between patients with and without hepatitis B virus infection. Int J Infect Dis.

[23] Delgrange E, Donckier JE (2000). Cryptococcal meningitis and Cushing's syndrome. Lancet.

